# Metabolite-based clustering and visualization of mass spectrometry data using one-dimensional self-organizing maps

**DOI:** 10.1186/1748-7188-3-9

**Published:** 2008-06-26

**Authors:** Peter Meinicke, Thomas Lingner, Alexander Kaever, Kirstin Feussner, Cornelia Göbel, Ivo Feussner, Petr Karlovsky, Burkhard Morgenstern

**Affiliations:** 1 Department of Bioinformatics, Institute of Microbiology and Genetics, University of Göttingen, Göttingen, Germany; 2 Department of Developmental Biochemistry, Institute for Biochemistry and Molecular Cell Biology, University of Göttingen, Göttingen, Germany; 3 Department for Plant Biochemistry, Albrecht-von-Haller-Institute for Plant Sciences, University of Göttingen, Göttingen, Germany; 4 Molecular Phytopathology and Mycotoxin Research Unit, University of Göttingen, Göttingen, Germany

## Abstract

**Background:**

One of the goals of global metabolomic analysis is to identify metabolic markers that are hidden within a large background of data originating from high-throughput analytical measurements. Metabolite-based clustering is an unsupervised approach for marker identification based on grouping similar concentration profiles of putative metabolites. A major problem of this approach is that in general there is no prior information about an adequate number of clusters.

**Results:**

We present an approach for data mining on metabolite intensity profiles as obtained from mass spectrometry measurements. We propose one-dimensional self-organizing maps for metabolite-based clustering and visualization of marker candidates. In a case study on the wound response of *Arabidopsis thaliana*, based on metabolite profile intensities from eight different experimental conditions, we show how the clustering and visualization capabilities can be used to identify relevant groups of markers.

**Conclusion:**

Our specialized realization of self-organizing maps is well-suitable to gain insight into complex pattern variation in a large set of metabolite profiles. In comparison to other methods our visualization approach facilitates the identification of interesting groups of metabolites by means of a convenient overview on relevant intensity patterns. In particular, the visualization effectively supports researchers in analyzing many putative clusters when the true number of biologically meaningful groups is unknown.

## Background

Metabolomics is a fundamental approach in basic research to detect and quantify the low molecular weight molecules (metabolites) in a biological sample. Besides the other so-called "omics" technologies (genomics, transcriptomics, proteomics), metabolomics is becoming a key technology that facilitates the measurement of the ultimate phenotype of an organism [1]. In particular, metabolomics allows undirected global screening approaches based on the measurements of signal intensities for a large number of intracellular metabolites under varying conditions, such as disease or environmental and genetic perturbations [2-8]. In order to identify relevant metabolites in terms of indicative metabolic markers, it is essential to provide tools for exploratory analysis of metabolome data generated by high-throughput analytical measurements [9,10]. For instance, the analysis of complex mass spectrometry data can cover relative intensities for a large number of metabolites under different conditions and requires advanced data mining tools to study the corresponding multivariate intensity patterns.

**Figure 1:**
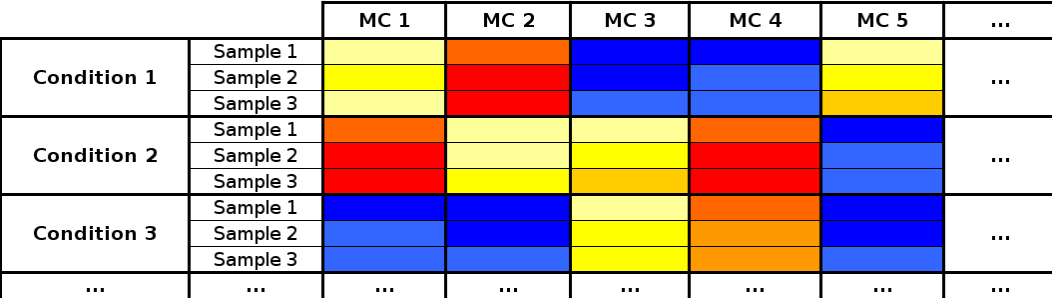
**Illustration of differences between sample-based clustering and metabolite-based clustering**. A toy example matrix of intensity measurements as obtained from LC/MS experiments. The horizontal dimension corresponds to metabolite (or marker) candidates. The vertical dimension corresponds to conditions and repeated measurements within these conditions. A row represents a sample for sample-based clustering, while a column corresponds to a (putative) metabolite for metabolite-based clustering. Colors represent different intensity values.

Clustering of intensity profiles from mass spectrometry measurements is an unsupervised approach to analyze metabolic data. In analogy to clustering of gene expression data [11], one may distinguish between sample-based clustering and metabolite-based clustering. In the latter case, the assumption is that metabolites sharing the same profile of accumulation or repression under a given set of conditions are likely to result from the same biosynthetic pathway or possibly are part of the same regulatory system. In that way, metabolite-based clustering parallels the gene-based clustering of expression data, where groups of similar expression profiles may indicate co-regulated genes [11]. In metabolite-based clustering, the intensities of a metabolite under certain experimental conditions provide an intensity vector representation for multivariate analysis. Metabolite-based clustering usually yields a large number of vectors (metabolite candidates) with comparably few dimensions (conditions). In contrast, sample-based clustering implies only few intensity vectors according to the number of conditions and repetitions. In turn, the dimensionality of these vectors is large, according to the number of (putative) metabolites. Thus, the two clustering approaches correspond to different views on a given matrix of intensity measurements (see figure 1): in one case the data vectors for multivariate analysis are derived from rows (samples in figure 1), in the other case vectors are derived from columns (metabolite candidates in figure 1). While repetition of measurements is essential for sample-based clustering, for metabolite-based clustering it is desirable but not strictly necessary, depending on the quality of data underlying the analysis.

Regarding the scope of application, sample-based clustering for unbiased, comprehensive metabolite analysis is often applied in order to identify different phenotypes [12]. In other cases, phenotypes are known and supervised methods may be applied to identify discriminative metabolic markers [1,13]. In contrast, the objective of metabolite-based clustering is to identify biologically meaningful groups of markers. The common approach is to combine dimensionality reduction and clustering methods: First, a sample-based principal component analysis (PCA) is performed to compute a subset of principal components. Then the metabolite-specific PCA loadings of these components are used for metabolite-based clustering using K-means [6] or hierarchical methods [14]. In these cases, the choice of experimental setup usually suggests a certain number of clusters which considerably facilitates the analysis. However, for a complex setup with several possibly overlapping conditions it is difficult to make assumptions about the number of relevant clusters. Therefore, metabolite-based clustering also requires suitable tools for visual exploration as an intuitive way to incorporate prior knowledge into the cluster identification process.

Here we introduce an approach to metabolite-based clustering and visualization of large sets of metabolic marker candidates based on self-organizing maps (SOMs). Unlike applications of the classical two-dimensional SOMs, we are proposing one-dimensional linear array SOMs (1D-SOMs). The 1D-SOM supports the search for relevant metabolites in two aspects: First, according to the assignment of data vectors to certain array positions, a "pre-clustering" of the data facilitates the analysis of large and noisy data sets. The resulting clusters provide building blocks for biologically meaningful groups of markers. In general, the determination of relevant groups requires task-specific knowledge in order to aggregate related clusters or to discard "spurious" clusters which cannot be associated with any biological meaning. This second step is supported by the dimensionality-reduced representation which results from the mapping to the linear array. By means of this mapping, 1D-SOMs allow to visualize the variation of intensity patterns along the array axis. This visualization provides a quick overview on relevant patterns in large data sets and facilitates the aggregation of related neighboring clusters. In particular, this kind of visual partitioning provides a powerful means to cope with the problem of an unknown number of "true" clusters which in general cannot be solved without task-specific constraints [15]. In the same way, spurious clusters, which do not represent any relevant groups, can easily be identified by visual inspection.

## Clustering and Visualization of Metabolite Candidates

The objective of our approach is to provide a convenient visual overview on potential metabolite clusters across a sample set of marker candidates. A marker candidate is characterized by its intensity profile under certain conditions. Thus, the marker can be represented by some *d*-dimensional vector **x **which contains the condition-specific quantities as inferred from mass spectrometry intensities. Besides the intensity profile vector **x**_*i*_, also a particular retention time (rt) index and mass-to-charge ratio (*m/z*) is associated with each marker candidate *i *in a given sample. While the intensity profiles are used in the clustering algorithm as shown below, the rt and *m/z *indices are only used for interpretation of the resulting groups (see section "visualization").

### Normalization

In general, mass spectrometry-based metabolite profiling is performed for each condition with multiple samples. For clustering, we use average intensity values of replicas for each marker candidate and treatment condition. After the averaging step, each marker candidate is represented by a vector with *d *dimensions corresponding to *d *experiment conditions. The averaging is important in order to compensate for random variations between different measurements and can be viewed as a noise reduction step. In principle, repeated measurements for averaging are not strictly necessary for application of our clustering approach. In practice, however, the noise reduction will help to achieve reproducible results. Furthermore, repeated measurements allow to evaluate the robustness of the clustering: single replica samples may be left out to analyze the variation induced by this kind of "leave-one-out" disturbance. In other words, it becomes possible to measure clustering or prototype stability with respect to a reduced quality of the training data. As compared with a marker-based cross-validation which reduces the size of the training set due to left out markers, the sample-based cross-validation allows to detect the same groups of markers across all leave-one-out folds.

In order to improve the comparability between putative metabolites of different abundance, the vector of intensity values for each marker candidate is normalized to Euclidean unit length. The normalization step ensures that marker clustering only depends on relative intensities and not on the usually large differences of absolute intensities. Therefore, the normalization allows to detect related metabolites irrespective of their abundancies. Without normalization, the clustering would mainly reflect the length variation within the set of marker candidate vectors.

### Topographic Clustering

In our 1D-SOM algorithm, a particular cluster arises from a group of marker candidates assigned to one of K "prototype" vectors **w**_*k *_∈ ℝ^*d *^for *k *= 1,..., *K*. A prototype vector corresponds to an average intensity profile and can be viewed as a noise-reduced representation of the associated marker candidates in that group. The clustering algorithm imposes a topological order on the prototypes according to a one-dimensional linear array. In that way, the projection onto an ordered set of prototypes also provides a dimensionality-reduced representation of the data in terms of a one-dimensional array index. The objective of the ordering is that prototypes adjacent in the array should provide more similarity than prototypes with distant array positions. The algorithm for optimization of prototypes is based on topographic clustering, which is a well-known technique in bioinformatics, usually applied by means of two-dimensional SOMs [16]. Unlike classical SOM applications, our one-dimensional map can be used to visualize the variation of intensity profiles along the array of prototypes within a common 2D color or gray level image (see next section).

For optimization of prototypes we utilize the principle of topographic vector quantization [17], which corresponds to the SOM learning scheme discussed in [18]. Our realization provides a stable and robust algorithm which only requires little configuration effort. The only parameters which may require modification of default values are the number of prototypes (array length) and the minimal amount of prototype smoothing. While the number of prototypes corresponds to the maximal number of clusters, the smoothing parameter controls the similarity of nearby prototypes. Smoothing is achieved by using confusion probabilities *h*_*jk *_which model the similarity of two prototypes **w**_*j*_, **w**_*k*_. The indices *j*, *k *∈ {1,..., *K*} of the prototypes correspond to positions in a linear array where nearby positions (indices) imply high similarity. The confusion probabilities are computed from normalized Gaussian functions depending on the bandwidth parameter *σ *as follows:

$$h_{jk}=\frac{\exp\left(-\frac{1}{2\sigma^{2}}{\left(j-k\right)}^{2}\right)}{\sum_{l=1}^{K}\exp\left(-\frac{1}{2\sigma^{2}}{\left(j-l\right)}^{2}\right)}$$

It is important to note that the final number of clusters depends on both, the maximal number of prototypes *K *and the smoothing parameter *σ*. This means that for a large amount of smoothing (high *σ *value) the actual number of clusters can be much smaller than the number *K *of available prototypes. In particular for a sufficiently high degree of smoothing, some prototypes may associate with zero-size clusters, i.e. they do not represent actual clusters. These prototypes are merely influenced by neighboring prototypes, without assignment to marker data.

During optimization, the smoothing parameter *s *is decreased from a large initial value with a small number of resulting clusters towards a minimal final value with an increased number of groups. With this kind of "annealing" process one tries to avoid bad local minima of the objective function which may result in a disrupted order of prototypes. For each annealing step with a particular (fixed) *σ *the optimization is realized by minimization of an objective function which measures the squared distances between prototypes and intensity data vectors. The objective function depends on the matrix **X **of *N *intensity column vectors **x**_*i*_, a matrix **W **of *K *prototype column vectors **w**_*j *_and an *N *× *K *matrix **A **of binary assignment variables *a*_*ij *_∈ {0, 1}. If *a*_*ij *_= 1, then data vector **x**_*i *_is exclusively assigned to the *j*-th prototype. For a fixed *σ *the following objective function is minimized in an iterative manner:

$$E_{\sigma }(X,A,W)=\sum_{i}\sum_{j}a_{ij}\sum_{k}h_{jk}{\left\|x_{i}-w_{k}\right\|}^{2}$$

The minimization iterates two optimization steps until convergence: first for given prototypes all assignment variables are (re)computed according to:

$$a_{ij}=\{\begin{array}{ll} 1 & \text{if }j=\arg\underset{l}{\min}\sum_{k}h_{lk}{\left\|x_{i}-w_{k}\right\|}^{2}\\ 0 & \text{else} \end{array},$$

Then the prototype vectors are (re)computed according to:

$$w_{k}=\frac{\sum_{i}\sum_{j}a_{ij}h_{jk}x_{i}}{\sum_{l}\sum_{m}a_{lm}h_{mk}}$$

The overall optimization scheme also involves a prior initialization step for the matrix **W **of prototypes and an annealing schedule for the smoothing parameter *s*. For initialization, all prototypes (columns of **W**) are placed along the first principal component axis within a small interval around the global mean vector. The annealing schedule is chosen to realize an exponential decrease of *σ *over 100 steps, starting with a maximum value *σ*_max _= 100 and ending with an adjustable minimum value which we set to *σ*_min _= 0.1. In supplementary material (see Additional file 1) a video clip shows the annealing process for the experimental data that is used in our case study (see section "Case study for experimental evaluation"). In our experiments, the (deterministic) annealing has shown to provide an efficient strategy to find deep local minima of the objective function. In particular, we found that it ensures good reproducibility of results because it makes the approach robust with respect to the initialization of prototypes. In all cases we observed that, besides the above principal component initialization, also different random initializations resulted in exactly the same prototypes up to a possibly reversed order. This behaviour can be explained by the fact that for a sufficiently high smoothing parameter the resulting 1D-SOM corresponds to a "dipole" where the ends (first and last prototype) provide the only non-zero size clusters (see Additional file 1). In this case, the line segment between these two prototypes is approximately collinear to the first principal component axis.

### Visualization

The result of the marker clustering process is an ordered array of prototypes in terms of a one-dimensional self-organizing map (1D-SOM) as described in the previous section. Each prototype represents a group of marker candidates and corresponds to an average intensity profile of that group. Therefore, the prototype-specific intensity profile can be viewed as a noise-reduced representation of all marker candidates assigned to this prototype. The order of prototypes in the array implies that similar intensity profiles are closer to each other than unrelated intensity profiles.

**Figure 2:**
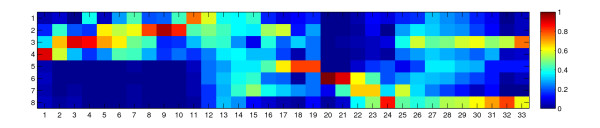
**Visualization of one-dimensional self-organizing map after clustering**. 1D-SOM matrix after metabolite-based clustering with 33 prototypes. The horizontal and vertical dimensions correspond to prototypes and experimental conditions, respectively. The color of matrix elements represent (average) intensity values according to the color map on the right hand side.

1D-SOMs are well-suitable for visualization and interpretation of multivariate data. Figure 2 shows a color-coded 1D-SOM of metabolomic data from LC/MS measurements (see also section "Results and Discussion"). The horizontal dimension of the matrix corresponds to the dimension of the SOM, i.e. the linear array axis. Each column of the matrix represents the intensity profile of one prototype. A prototype represents a group of markers (cluster) assigned to the corresponding array position. The vertical dimension corresponds to the experiment-specific conditions. In our example eight conditions were used, therefore the matrix consists of eight rows. The color coding of a matrix element represents the intensity value associated with a prototype and a particular experimental condition. The color corresponds to intensity values according to a so-called "jet map", i.e. blue and red elements represent low and high intensity values, respectively.

The 1D-SOM matrix in figure 2 shows the resulting 33 prototypes that have been optimized during the clustering process in our case study (see section "Case study for experimental evaluation"). The figure reveals a certain block structure of the prototype matrix which can be perceived as a visual partitioning along the linear array axis. Within the corresponding blocks, the prototypes are very similar or they show gradual changes ("trends") of a certain intensity pattern. For example, prototypes 18 and 19 show a unique pattern which indicates, that metabolite candidates in the corresponding two clusters provide a significantly higher intensity under the fifth condition than under the remaining seven conditions. If conditions correspond to time points, as in the example, the "highlighting" of a specific condition usually indicates the presence of so-called "transient" markers. On the other hand, blocks of putative markers may result from more complex intensity patterns, e.g. when related prototypes show high intensity values for several "overlapping" conditions simultaneously. In particular, a smooth variation of a pattern along a block may indicate a time course or trend, for instance metabolite concentration under temporal development. In figure 2, overlapping conditions can especially be observed among the first twelve prototypes which show a continuous time-dependent evolution of the intensity pattern. However, prototypes 11 and 12 show an intensity maximum for the (first) control condition and therefore should be assigned to a separate block (see section "Application of 1D-SOMs"). In general, prior knowledge about reasonable condition overlaps within the experimental setup is necessary to identify meaningful blocks of prototypes.

**Figure 3:**
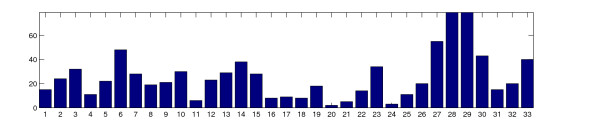
**Bar plot of cluster sizes**. Bar plot of size for all clusters associated with the 33 prototypes. The horizontal and vertical dimensions correspond to prototype number and cluster size, respectively. The height of a prototype-specific bar is proportional to the number of marker candidates assigned to this prototype.

**Figure 4:**
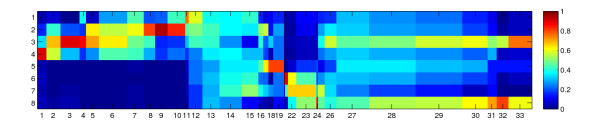
**Visualization of one-dimensional self-organizing map according to cluster size**. Alternative view of 1D-SOM matrix after metabolite-based clustering with 33 prototypes. The horizontal and vertical dimensions correspond to prototypes and experimental conditions, respectively. The color of matrix elements represents (average) intensity values according to the color map on the right hand side. The width of the matrix column for each prototype is proportional to the number of marker candidates assigned to this prototype.

Figure 3 shows a bar plot that displays the number of marker candidates associated with each prototype. This kind of histogram measures the density of candidates along the linear array axis and may provide additional evidence for a particular block partitioning. In this case a block usually shows a local density maximum (mode) bordered with distinct minima. Figure 4 shows a variant of the 1D-SOM matrix visualization which combines the prototype intensity profile and cluster size information. Here, the width of each column is proportional to the cluster size. This representation facilitates the identification of large clusters, while spurious clusters are usually suppressed in the corresponding visualization.

**Figure 5:**
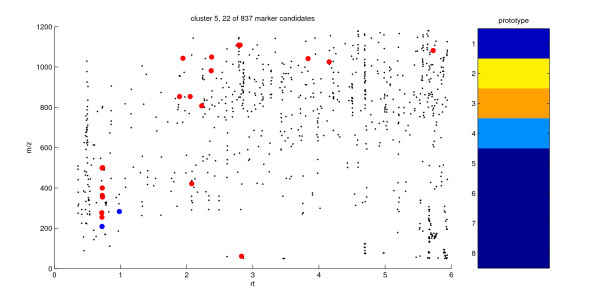
**rt-m/z plot of cluster 5**. Scatter plot in the rt-*m/z *plane for identification of adducts and unknown marker candidates. Marker candidates associated with prototype 5 are prepresented as big red dots in the retention time vs. mass-to-charge ratio (rt-*m/z*) plane. The wound markers represented by the big blue dots are JA (*m/z *209, rt 0.72 min) and OPC-4 (formate adduct, *m/z *283, rt 0.98 min). The marker candidates that are in a vertical line with the blue dot at rt 0.72 min exhibit a noticeable vertical stack. The remaining marker candidates of the experiment are represented by small black dots. The average intensity profile associated with prototype 5 is shown on the right hand side.

**Figure 6:**
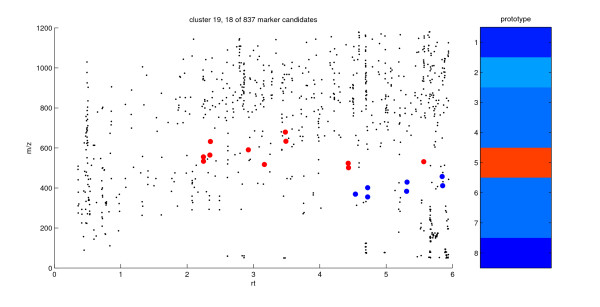
**rt-m/z plot of cluster 19**. Marker candidates associated with prototype 19 as big red dots in the retention time vs. mass-to-charge ratio (rt-*m/z*) plane. The markers represented by the big blue dots are COOH-22:0, OH-22:0, OH-24:0 and OH-26:0 (see also table 2) and the formate adducts of the latter three hydroxy fatty acids These formate adducts are characterized by identical rt values and a mass shift of *m/z *46. The remaining marker candidates of the experiment are represented by small black dots. On the right hand side the average intensity profile associated with prototype 19 is shown.

Figures 5 and 6 visualize particular clusters by means of a scatter plot in the retention time vs. mass-to-charge ratio plane (rt-*m/z *plot). Big red dots correspond to marker candidates associated with the particular prototype and small black dots correspond to the remaining marker candidates of the same data set. The rt-*m/z *plot complements the 1D-SOM visualization of intensity profiles and shows an overview of those prototype-specific marker properties that are not used for the intensity-based clustering. In this plot, the distribution of marker candidates of a particular group within the rt-*m/z *plane can be analyzed. For example, vertical stacks of marker candidates may indicate adducts of particular compounds since the corresponding markers do not differ in retention time.

## Case study for experimental evaluation

The objective of our experimental evaluation is not to provide "hard" performance indices, e.g. in terms of detection rates, but rather to show how our 1D-SOM approach can support scientists in the interpretation of large metabolic data sets, especially for the identification of interesting groups of markers. On one hand there is no "benchmark" data set with known markers available which provides a complex experimental setup with a sufficiently large number of conditions. On the other hand our 1D-SOM approach is designed for visual exploration of multivariate marker data which is difficult to evaluate in terms of a simple performance criterion. Therefore, we here provide a case study in order to illustrate the practical utility of our method. For that purpose we chose a well-established experimental setup for analyzing the wound response of plants.

**Figure 7:**
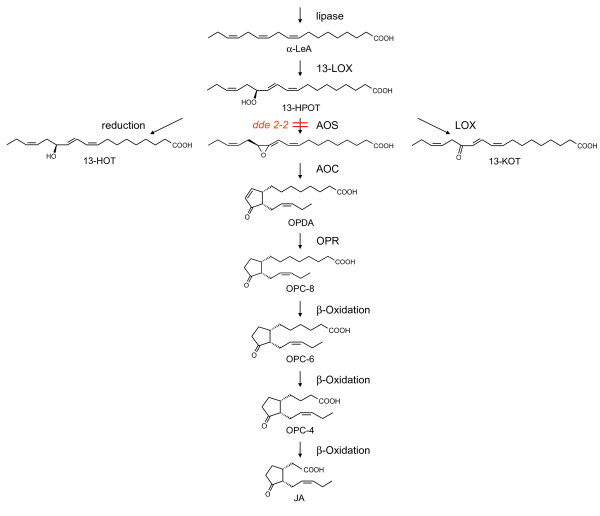
**Oxylipin biosynthesis**. Oxylipin biosynthesis starts with the release of *α*-linolenic acid (*α*-LeA) from chloroplast membranes [21]. This fatty acid can be metabolized by the action of 13-lipoxygenase (13-LOX) that leads to (13*S*)-hydroperoxyoctadecatrienoic acid (13-HPOT). The first step in jasmonic acid (JA) biosynthesis is carried out by an allene oxide synthase (AOS) leading to an unstable allene oxide. This intermediate is converted by an allene oxide cyclase (AOC) into (9*S*,13*S*)-12-oxo phytodienoic acid (OPDA). The subsequent step, reduction of the cyclopentenone ring, is catalysed by an OPDA reductase (OPR). Three rounds of *β*-oxidative side-chain shortening starting with 3-oxo-2-(pent-2'-enyl)-cyclopentane-1-octanoic acid (OPC-8) via 3-oxo-2-(pent-2'-enyl)-cyclopentane-1-hexanoic acid (OPC-6) and 3-oxo-2-(pent-2'-enyl)-cyclopentane-1-butanoic acid (OPC-4) lead to the synthesis of JA. Beside the JA biosynthesis pathway, the LOX-product 13-HPOT can be either reduced to (13S)-hydroxyoctadecatrienoic acid (13-HOT) or under certain conditions, such as low oxygen pressure to 13-ketooctadecatrienoic acid (13-KOT) by the action of 13-LOX. The mutation of the AOS gene of the *dde *2–2 mutant leads to a deficiency in the JA biosynthesis [26].

Since plants are sessile organisms, they are directly exposed to environmental conditions. Therefore plants have developed special mechanisms to respond to injuries caused by herbivores, mechanical wounding and pathogen attack. Mechanical damage activates diverse mechanisms directed to healing and defense [19]. These processes include the generation of specific molecular signals that activate the expression of wound-inducible genes [20,21]. Until now the analysis of the wound response has primarily focused on the transcriptional response [22] and on a special set of metabolites involved in early signal transduction events. Here fatty acid derived signals, like jasmonic acid (JA) and its derivatives (referred to as jasmonates), as well as other oxygenated fatty acid metabolites (referred to as oxylipins) play a crucial regulatory role in mediating the wound response [19,23]. To show the potential of our 1D-SOM, we analyzed the metabolite profile of the thale cress *Arabidopsis thaliana *during a wounding time course. The genome of this model plant has been sequenced and its wound response is well characterized [20,24]. To describe the wound response of *A. thaliana *in a broad functional context we compared a wounding time course of wild type (wt) plants with that of *dde *2–2 mutant plants. The *dde *2–2 plants are deficient in JA biosynthesis due to the mutation of the *allene oxide synthase *(*AOS*) gene (see figure 7). In wt plants, the encoded enzyme catalyzes the first committed step in JA biosynthesis [25].

Because the wound response shows a complex network of integrated biochemical signals we used an unbiased metabolomic analysis to extend our knowledge on global metabolic changes at early time points after wounding. In contrast to targeted procedures, this type of analysis is able to cope with complex metabolic situations in a more realistic and global way by including many metabolites that are unknown so far but are regulated in a certain context. For the interpretation of data sets of such high complexity, advanced data mining tools are essential.

### Plant growth and wounding

Two plant lines were used: wt plants of *A. thaliana *(L.) ecotype Columbia-0 (Col-0) and the JA-deficient mutant plants *dde *2–2 [26]. Plants were grown on soil under short day conditions. Rosette leaves of eight-week-old plants were mechanically wounded using forceps [27]. Whole rosettes of unwounded plants (control, 0 h) and wounded plants (0.5, 2 and 5 hours post wounding (hpw)) were harvested and immediately frozen in liquid nitrogen. To minimize biological variation, rosettes of five to ten plants were pooled for each time point.

### Experimental setup

The data set resulting from the wounding experiment consists of eight conditions (see Table 1). The first four conditions reflect the metabolic situation within a wounding time course of wt plants starting with the control plants followed by the plants harvested 0.5, 2 and 5 hpw. The conditions 5 to 8 represent the same time course for the JA deficient mutant plant *dde *2–2.

**Table 1:** Experimental conditions for wounding of A. thaliana wild type (wt) and dde 2–2 mutant (dde 2–2) plants.

A. thaliana Col-O	hour post wounding (hpw)	condition	sample name
wt	0	1	wt, 0 h
	0.5	2	wt, 0.5 hpw
	2	3	wt, 2 hpw
	5	4	wt, 5 hpw
*dde *2–2	0	5	*dde *2–2, 0 h
	0.5	6	*dde *2–2, 0.5 hpw
	2	7	*dde *2–2, 2 hpw
	5	8	*dde *2–2, 5 hpw

### Metabolite extraction and measurement

Plant material was homogenized under liquid nitrogen and subsequently extracted using methanol/chloroform/water (1:1:0.5, v:v:v) as described in [28], but without adding internal standards. Four independent extractions were performed for each condition.

The chloroform phase containing lipophilic metabolites was analyzed by Ultra Performance Liquid Chromatography (ACQUITY UPLC™ System, Waters Corporation, Milford) coupled with an orthogonal time-of-flight mass spectrometer (TOF-MS, LCT Premier™, Waters Corporation, Milford) working with negative electrospray ionization (ESI) in an *m/z *range of 50 to 1200. For chromatographic separation an ACQUITY UPLC™ BEH SHIELD RP18 column (1 × 100 mm, 1.7 *μ*m, Waters Corporation, Milford) was used with a methanol/acetonitrile/water gradient, containing 0.1% (v/v) formic acid. The LC/MS analysis was performed at least twice for each extract resulting in nine replicas for each condition. The identification of metabolites was verified by exact mass measurement and coelution with authentic standards.

### Data processing

The raw mass spectrometry data of all samples were processed (deconvolution, alignment, deisotoping and data reduction) using the MarkerLynx™ Application Manager for MassLynx™ software (Waters Corporation, Milford) with parameter settings as shown in the supplementary table "MarkerLynx parameters" (see Additional file 2). MarkerLynx™ automatically performs a noise reduction which results in zero values for certain low intensity peaks. The processing resulted in 6048 marker candidates.

Unsupervised methods for metabolite-based clustering strongly rely on marker quality. The quality mainly depends on reproducibility and biological interpretability. Without prior selection, large amounts of non-informative markers with little intensity variation across different conditions would dominate the clustering results and complicate further analysis. In general, number and quality of selected markers should depend on the specific requirements of a particular study. Therefore, a task-dependent trade-off between number and quality of marker candidates has to be found. In our case we performed a Kruskal-Wallis test [29] on the intensities of each marker candidate and used the corresponding p-value as a measure of quality. Considering the rank order of marker candidate intensities, this non-parametric test can be used to detect significant variation of the condition-specific mean ranks. In that way we selected a subset of high-quality markers using a conservative confidence threshold of 10^-6^. The selection contained 837 marker candidates with a p-value below the specified threshold (see Additional file 3 for CSV file of data set).

## Results and Discussion

In the following we first present the results of our case study using the proposed 1D-SOM algorithm. Then we apply hierarchical clustering analysis (HCA) in combination with the K-means algorithm [15] and finally principal component analysis (PCA) for comparison. For implementation of the 1D-SOM training and visualization we used the MATLAB^® ^programming language together with the Statistics Toolbox^® ^for HCA and K-means clustering.

### Application of 1D-SOMs

Because the true number of biologically meaningful groups is unknown, we had to choose a sufficiently high number of prototypes for clustering. In accordance with a prior robustness study (see section "Accessing Robustness") we chose *K *= 33 prototypes for the analysis in our case study. For higher numbers of prototypes we observed an increasing number of singleton clusters as well as the occurrence of "empty" clusters without any assigned marker candidates.

First, the resulting 1D-SOM allows an overview of the complex metabolic situation within the sample set of examination (see figures 2 and 4). Simultaneously, a more specific analysis of distinct clusters can be performed by means of rt-*m/z *scatter plots (see figures 5 and 6). In figure 2, the 1D-SOM of the time course of the wound experiment including wt and *dde *2–2 mutant plants is shown. To our knowledge, this is the first visualization that shows a convenient overview of the intensity patterns of several hundred marker candidates of the lipophilic fractions. The intensity profiles of these 837 lipophilic marker candidates are represented by 33 prototypes. The visualization clearly reveals the existence of different blocks of intensity patterns.

A first dominant block (block A, see figure 2 and table 2) consists of the prototypes 1 to 10. The block contains 250 marker candidates, which accumulate in wt plants after wounding (condition 2–4) but are either missing or show very low intensities in the *dde *2–2 mutant plants (condition 6–8). Within block A a remarkable shift of late enriched marker candidates (prototype 1) over time stable candidates (prototypes 5–7) towards very early enhanced and transient marker candidates (prototype 9) can be observed. Thus, block A represents candidates that are characteristic for the wound response of wt plants and which clearly show a trend along the first 10 prototypes of the 1D-SOM.

**Table 2:** Formation of blocks based on the interpretation of prototype profiles and identification of corresponding markers.

Block	Prototypes	# markers	Marker characteristics	Identified wound markers	Prototype
A	01 – 10	250	Accumulation in wild type plants after wounding	JA-Ile (*m/z *322)	9
				dn-OPDA (*m/z *263)	8
				OPC-4 (formate adduct, *m/z *283)	5
				JA (*m/z *209)	5
				OPDA (*m/z *291)	2
				OH-JA-Ile (*m/z *338)	1
				OH-JA (*m/z *225)	1
				COOH-JA-Ile (*m/z *352)	1
B	11 – 12	29	Accumulation in wt control plants	--	--
C	13 – 17	112	Mainly indifferent	--	--
D	18 – 19	26	Accumulation in mutant control plants	COOH-22:0 (*m/z *369)	19
				OH-22:0 (*m/z *355)	19
				OH-24:0 (*m/z *383)	19
				OH-26:0 (*m/z *411)	19
E	20 – 24	58	Accumulation in mutant plants after wounding	HHT (*m/z *265)	21
				HOT (*m/z *293)	22
				KOT (*m/z *291)	22
F	25 – 33	362	Delayed accumulation in mutant plants after wounding	--	--

Prototypes 20–24 can be grouped in a block E (see figure 2 and table 2). This rather small block contains 58 marker candidates typical for the wound response in the JA deficient *dde *2–2 mutant plants and, thus, acts as a counterpart of block A. In wt plants block E marker candidates are either missing or show very low intensities. Within block E a shift from very early transient marker patterns (prototype 20) over very early time-stable patterns (prototype 21 and 22) towards late marker patterns of the wound response (prototype 24) is obvious.

A very small but remarkable block consists of prototypes 18 and 19 (block D, see figure 2 and table 2). Here 26 marker candidates accumulate in non-treated plants of the *dde *2–2 mutant but not in non-treated wt plants. Within 0.5 hpw the level of these candidates decreased in *dde *2–2 mutant plants. Therefore, block D represents marker candidates down regulated during the wound response in *dde *2–2 mutant plants. Surprisingly, there is a dominating block summarizing 362 marker candidates with increasing intensities both in wt and in mutant plants after wounding (block F, prototypes 25 to 33, see figure 2 and table 2). The visualization revealed that the accumulation of these putative metabolites started earlier in wt plants (2 hpw) when compared to the mutant plants (5 hpw). The wound marker candidates of block F seem to be regulated independently from the JA pathway.

Block A and D are interrupted by a block B summarizing marker candidates that accumulate in wt control plants (prototype 11 and 12) and block C showing mainly indifferent intensity patterns (prototype 13–17). After the initial assignment of prototypes, blocks were analyzed in more detail at the level of individual metabolites. For this purpose we searched the data set for well known metabolic constituents of the wound response, such as JA, its immediate precursors 12-oxo-phytodienoic acid (OPDA), 3-oxo-2-(pent-2'-enyl)-cyclopentane-1-octanoic acid (OPC-8), 3-oxo-2-(pent-2'-enyl)-cyclopentane-1-hexanoic acid (OPC-6) and 3-oxo-2-(pent-2'-enyl)-cyclopentane-1-butanoic acid (OPC-4), as well as JA derivatives and the roughanic acid-derived homolog of OPDA, dn-OPDA (see also figure 7) [23,30]. By this approach, eight known wounding markers could be identified in block A (see figure 2 and table 2). Markers related to the wound response in the *dde *2–2 mutant plants are located in block D and E (see figure 2 and table 2). The JA-independent marker candidates of block F will be subject of further investigations.

#### Prototypes of block A represent wound markers of wt plants

As expected from the current literature on targeted and untargeted metabolic analysis [23,31,32], a significant number of wounding markers was identified exclusively in wt plants.

The wound markers JA (*m/z *209) and OPC-4 (formate adduct, *m/z *283) were detected in cluster 5 (see table 2). As visible in the rt-*m/z *plane in figure 5, the blue-colored JA dot at rt 0.72 min shows the lowest *m/z *value within a noticeable vertical stack. Dots of this stack may partially represent ESI-specific adducts of JA, such as the formate adduct (*m/z *255, rt 0.72 min). Due to the high similarity of intensity profiles between a metabolite and its adducts, metabolites and their adducts are likely to be assigned to the same prototype. Thus, adducts are easy to detect within the same cluster by means of stack formation which results from identical retention times.

Interestingly, prototype 5 associates the intensity profile of JA and its precursor OPC-4 (blue dot at rt 0.98 min in the rt-*m/z *plane in figure 5) with the profile of a group of marker candidates of high molecular weight (*m/z *range from 800 to 1200) not identified up to now. However, the arrangement of these metabolites in the JA-containing cluster suggests them to play a role in wound response of wt plants. The wound markers dn-OPDA (*m/z *263) and jasmonoyl-isoleucine (JA-Ile, *m/z *322) were detected in cluster 8 and 9, respectively (see figure 2 and table 2). These prototypes are associated with marker candidates characterized by a very early and transient intensity maximum at 0.5 hpw.

Similar to prototype 5, prototype 9 also associates the intensity profile of a small, rather polar wound signal substance (JA-Ile) with the profile of a group of markers of high molecular weight (*m/z *range from 850 to 1020) and stronger lipophilic properties (rt range from 2.5 to 4 min) not identified with certainty up to now. Interestingly, the time-dependent order of prototypes in the 1D-SOM allows the prediction that JA-Ile and the associated group of marker candidates of high molecular weight in cluster 9 are more transiently regulated than the main wound marker JA located in cluster 5. Therefore, the group of compounds associated with JA-Ile appears to represent valuable candidates for further investigations into the network of wound signaling in *A. thaliana*.

Hydroxy-JA (OH-JA, *m/z *225) and the JA-Ile derivatives hydroxy-jasmonoyl-isoleucine (OH-JA-Ile, *m/z *338) and carboxy-jasmonoyl-isoleucine (COOH-JA-Ile, *m/z *352) are assigned to prototype 1. All three substances show an intensity profile typical for late-occurring wound responsive metabolites. OH-JA is a product of JA modification with the capability to counteract the JA signaling pathway [31]. The JA-OH intensity pattern coincides with the postulated counterregulatory function of OH-JA. Like OH-JA, the polar JA-Ile derivatives OH-JA-Ile and COOH-JA-Ile show a delayed wound response in comparison to JA-Ile and JA, an observation also described in [23]. The wound marker OPDA (*m/z *291, see figure 2 and table 2) was detected in cluster 2 and therefore OPDA also represents a late wound marker.

#### Prototypes of block E represent wound markers of *dde *2–2 mutant plants

In *dde *2–2 mutant plants the wound response is disturbed by the deletion of the AOS enzyme activity. Therefore, products of the wound signaling pathway upstream of the AOS reaction should be enriched and have therefore been expected in block E. Candidates for the accumulation of precursors are hydroperoxides and hydroxides of fatty acids as well as keto fatty acids [33]. We have identified hydroxy hexadecatrienoic acid (HHT, *m/z *265) in cluster 21 and hydroxy octadecatrienoic acid (HOT, *m/z *293) as well as keto octadecatrienoic acid (KOT, *m/z *291) in cluster 22, respectively (see table 2). These observations confirm our hypothesis that the intensity levels of all three metabolites (HHT, KOT and HOT) are regulated by the AOS enzyme activity.

#### Prototypes of block D represent markers accumulating in *dde *2–2 mutant control plants

Block D with prototypes 18 and 19 combines 26 marker candidates with intensity profiles indicating accumulation in the control plants of the *dde *2–2 mutant and a decrease after wounding of these plants. However, these candidates exhibit only low intensities and are not altered in intensity by wounding in wt plants (see figure 2).

The seven blue-colored markers of cluster 19 shown in figure 6 could be identified as very long chain dicarboxylic and hydroxy fatty acids so far not described in the context of plant wound responses (see table 2): docosanedioic acid (COOH-22:0, *m/z *369, rt 4.54 min), hydroxy-docosanoic acid (OH-22:0, *m/z *355, rt 4.72 min), hydroxy-tetracosanoic acid (OH-24:0, *m/z *383, rt 5.31 min), hydroxy-hexacosanoic acid (OH-26:0, *m/z *411, rt 5.85 min) and the formate adducts of the latter three hydroxy fatty acids. These formate adducts are characterized by identical retention times and a mass shift of *m/z *46 regarding the molecular ion. The formation of strong formate adducts for the hydroxy fatty acids but not for the dicarboxylic fatty acid could be confirmed by LC/MS analysis of the corresponding standards. The analysis shows the potential of adduct formation occurring in ESI-MS analysis for the further identification of markers. Here the visualization by means of rt-*m/z *scatter plots makes it possible to recover specific adduct formation (see figure 6). Finally, the occurrence of these four very long chain dicarboxylic and hydroxy fatty acids in one cluster suggests that these metabolites are part of the same regulatory context.

### Application of HCA/K-means

For comparison of our 1D-SOM method with a more classical approach to clustering and visualization we performed hierarchical cluster analysis (HCA) in combination with K-means. The HCA/K-means scheme combines hierarchical clustering for prototype initialization with a K-means algorithm for iterative improvement of prototypes. For this purpose the resulting HCA dendrogram is cut at a particular distance to obtain a predefined number of ordered clusters. In the next step K-means is applied using the HCA partition means as initial prototypes.

**Figure 8:**
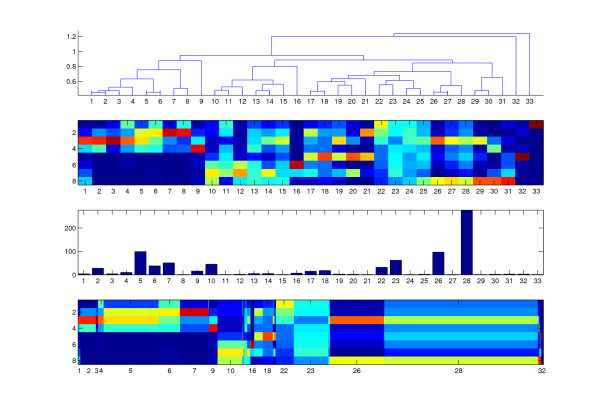
**Visualization of HCA/K-means results**. Visualization of results from hierarchical clustering combined with K-means with K = 33 prototypes. Top: pruned average linkage HCA dendrogram (vertical axis represents Euclidean distance). Second row: resulting K-means prototype vectors (vertical axis: conditions). Third row: bar plot of the corresponding cluster sizes (vertical axis: cluster size). Fourth row: scaled prototypes with width according to cluster size.

For direct comparison with the previous 1D-SOM results we performed an average linkage HCA/K-means clustering with 33 prototypes using Euclidean distances. Figure 8 shows the pruned HCA dendrogram, the resulting K-means prototype vectors, a histogram of the corresponding cluster sizes, and the scaled prototypes with width according to cluster size. The dendrogram by itself cannot be interpreted in terms of intensity profiles. In contrast to the 1D-SOM, the prototypes are only weakly ordered, which complicates the aggregation to meaningful blocks and the identification of interesting clusters (see figure 8, second row). The wound-induced marker candidates of *dde *2–2 mutant plants, for example, are mainly associated with prototypes 10, 12, 16 and 31, while the marker candidates which show accumulation in mutant control plants are distributed among cluster 18 and 32. Furthermore, eight clusters only contain a single marker candidate. These singleton clusters do not provide information about groups of related candidates sharing the same distinct intensity profile. Due to the weak prototype ordering it usually makes no sense to merge these singletons with neighboring clusters.

### Accessing Robustness

**Figure 9:**
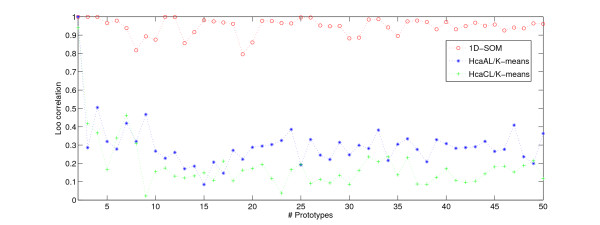
**Leave-one-out correlation of 1D-SOM vs. HCA/K-means**. Measuring robustness in terms of the leave-one-out (Loo) correlation of 1D-SOM in comparison with average linkage HCA/K-means (HcaAL/Kmeans) and complete linkage (HcaCL/Kmeans) for different numbers of prototypes.

To investigate the robustness of the cluster-based visualization approaches we applied the leave-one-sample-out strategy as motivated in section "Normalization". In that way we measured the robustness with respect to a reduced number of replicas: we removed one sample for each condition from the data and compared the resulting prototypes with the original array of prototypes obtained with the full data set with all nine samples per condition. In particular, we measured the Pearson correlation between the ordered prototype intensities of both arrays. We chose the reversed order of the original array if it yielded a higher correlation. As a measure of reproducibility, we took the mean correlation over the nine folds of the leave-one-out procedure. The mean leave-one-out correlation was computed for a varying number of prototypes, according to *K *= 2, 3,..., 50. The resulting curve plots in figure 9 clearly show that the 1D-SOM visualization approach is robust with respect to the simulated data quality loss. The 1D-SOM shows high stability of the prototype array under the induced disturbances: in most cases the correlation is above 0.9 with a mean of 0.947. In contrast, the correlations of the HCA/K-means approach are rather low with a mean of 0.299 for the average linkage variant. Using complete linkage instead of average linkage, the results (see figure 9) become even worse, as indicated by a mean correlation of only 0.184. These findings indicate that the "weak" prototype ordering of HCA/K-means, which results from the dendrogram structure, is not robust with respect to changing data quality. In particular, the lacking robustness can be observed for higher numbers of prototypes. Note that maximization of the correlation cannot be used to select an optimal number of clusters because this selection would result in the smallest possible number of clusters with highest correlation obtained for the trivial single prototype solution. However, the resulting correlation curves (see figure 9) can be used to select a sufficiently large *K *from the set of local maxima. Considering these curves we chose *K *= 33 prototypes for the more detailed analysis described in the two previous sections.

### Application of PCA

**Figure 10:**
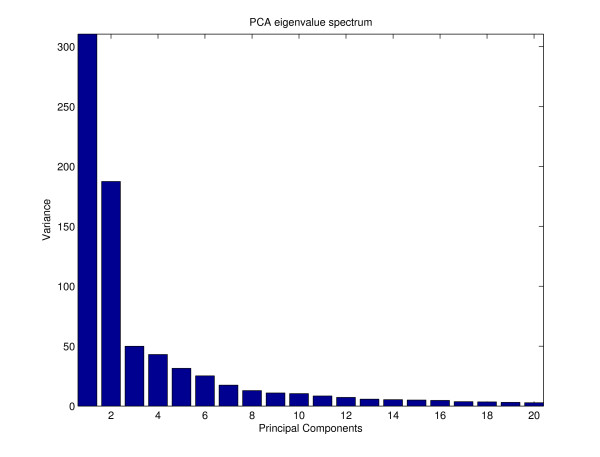
**Eigenvalue spectrum of sample-based PCA**. Eigenvalue spectrum of sample-based PCA showing variance of the first 20 principal components.

**Figure 11:**
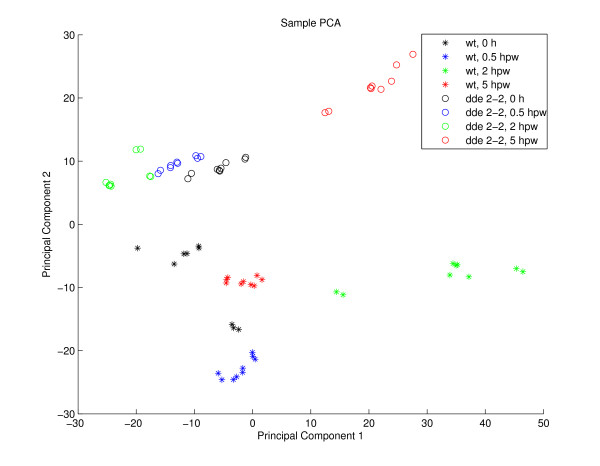
**Sample-based PCA scatter plot**. Visualization of experimental conditions according to the first two principal components of a sample-based PCA applied to the experimental data. Short identifiers for all experimental conditions are given on the right hand side. The abbreviations used in the legend are explained in table 1.

**Figure 12:**
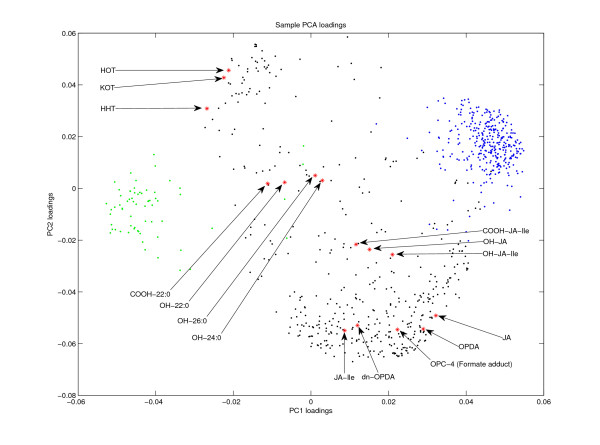
**Scatter plot of sample-based PCA loadings**. Visualization of PCA loadings for all marker candidates of the experiment. Loadings were calculated according to the first two principal components of sample-based PCA. Black, green and blue dots represent unidentified marker candidates. Green and blue dots correspond to candidates of clusters 14–15 and 27–33, respectively. Red asterisks represent identified markers. Marker abbreviations are explained in section "Application of 1D-SOM" and in table 2.

For comparison with the classical multivariate analysis approach, a PCA was performed on the samples of the dataset. PCA provides a linear dimensionality reduction with minimal loss of data variance. For this purpose the first eigenvectors of the estimated data covariance matrix (sorted by eigenvalues in descending order) serve as projection weights for the original data vectors. The reduced data coordinates (principal component scores) can be plotted in order to identify outliers or groups of correlated data samples. The corresponding eigenvector coordinates (loadings) can be used to identify clusters of correlated variables (marker candidates). The eigenvalues represent the amount of variance captured by the corresponding principal components. As a common preprocessing step, the marker-specific intensities (sample dimensions) were normalized to unit standard deviation before applying PCA. The eigenvalue spectrum (see figure 10) indicates that the first two principal components account for a large proportion of the total variance. The resulting plot of the first two principal component (PC) scores shows a clear phenotype separation of the eight conditions (see figure 11). The corresponding PCA loadings plot (see figure 12) contains two obvious clusters which mainly correspond to the marker candidates of cluster 14 and 15 in the 1D-SOM (green dots) and the marker candidates of cluster 27 to 33 (blue dots), respectively. The identified markers were tagged with the corresponding metabolite labels according to table 2. The plot shows a concentration of wound induced markers of wt plants in the "south east" quadrant and wound induced markers of *dde *2–2 mutant plants in the "north west" quadrant, respectively. However, there is no evidence for a more detailed cluster structure which could be inferred from the plot. The dicarboxylic and hydroxy fatty acid markers COOH-22:0, OH-22:0, OH-24:0 and OH-26:0 for example, share the same distinct intensity profile (see figure 2, prototype 19), but they do not seem to belong to a common cluster in the loadings plot. The lack of a simultaneous visualization of the corresponding intensity profiles complicates the interpretation of the plot substantially.

## Conclusion

We have introduced an approach to metabolite-based clustering for the identification of biologically relevant groups of metabolic markers in mass spectrometry data. Our algorithm is based on a special realization of one-dimensional self-organizing maps (1D-SOMs). In a case study about the wound response in *A. thaliana *we could show that our 1D-SOMs provide a visualization of multivariate marker data suitable for investigation of potential clusters. By means of a linear array of ordered prototypes the 1D-SOM representation gives a convenient overview on relevant patterns in complex multivariate data. Meaningful expected as well as unexpected clusters can be identified by visual inspection of the corresponding intensity profiles. In particular our approach supports the discovery of so far unknown markers on the basis of their location in the 1D-SOM array with respect to previously identified markers.

## Competing interests

The authors declare that they have no competing interests.

## Authors' contributions

PM implemented the clustering algorithm, drafted parts of the manuscript and contributed machine learning expertise, TL contributed conceptually and drafted parts of the manuscript, AK implemented the visualization and drafted parts of the manuscript, KF, CG and IF planned and generated the plant wound data set, analyzed the clustering results and drafted parts of the manuscript, PK contributed biological expertise and input to the concept of marker clustering, BM contributed conceptually. All authors read and approved the final manuscript.

## Supplementary Material

Additional file 1 Movie of the annealing process during clustering. The file cluster_process_33nodes.mpg contains a movie that shows the annealing process during clustering of the experimental data used in our case study. The annealing schedule realizes an exponential decrease of the smoothing parameter *σ *over 100 steps. The initial value is *σ*_*max *_= 100 and the final value is *σ*_*min *_= 0.1. Click here for file

Additional file 2 List of MarkerLynx™ parameters. The data file MarkerLynxParameters.xls contains an Microsoft^® ^Excel table with parameters that were used for data preprocessing with MarkerLynx™. Click here for file

Additional file 3 Table of marker candidates used in the case study. The data file dataset837.csv contains the marker candidates used for clustering and visualization. Rows correspond to particular marker candidates. The first column corresponds to marker candidate ID, the second and third column represent cluster ID and block ID according to table 2, respectively. The block IDs A, B, C, D, E and F are encoded by integers 1,..., 6. Columns 4 and 5 correspond to experimental nominal mass (*m/z*) and retention time (minutes), respectively. Columns 6 to 77 contain intensity values from mass spectrometry measurements. Here, nine successive values correspond to replicas of a particular experimental condition (see section "Case study for experimental evaluation"). The intensity values are ordered according to successive replicas for each condition (order of conditions according to table 1). Click here for file
